# Serum E-selectin and endothelial cell-specific Molecule-1 levels among people living with HIV on long term ART in Uganda: a pilot cross-sectional study

**DOI:** 10.1186/s12981-023-00519-x

**Published:** 2023-05-09

**Authors:** Andrew Weil Semulimi, Charles Batte, Alice Bayiyana, Mariam Nakabuye, John Mukisa, Barbara Castelnuovo, Rosalind Parkes Ratanshi, Bruce J Kirenga, Isaac Ssinabulya

**Affiliations:** 1 Lung Institute, Department of Medicine, College of Health Sciences, Makerere University, Kampala, Uganda; 2 Department of Immunology and Molecular Biology, School of Biomedical Sciences, College of Health Sciences, Makerere University, Kampala, Uganda; 3 Infectious Diseases Institute, Makerere University, Kampala, Uganda; 4 Department of Psychiatry, University of Cambridge, Cambridge, UK; 5 Department of Medicine, School of Medicine, College of Health Sciences, Makerere University, Kampala, Uganda

**Keywords:** E-Selectin, Endothelial activation, Long-term antiretroviral therapy

## Abstract

**Background:**

Prolonged exposure to HIV and anti-retroviral therapy (ART) has been linked with endothelial cell activation which subsequently predisposes people living with HIV (PLWH) to cardiovascular diseases. Serum biomarkers of endothelial cell activation such as E-Selectin and endothelial cell-specific molecule-1 (ESM-1) could aid in early detection of PLWH at a risk of cardiovascular diseases. However, there is a paucity of data on these biomarkers like E-selectin and endothelial cell-specific molecule-1 (ESM-1) among PLWH on long term ART (≥ 10 years) in Uganda. The aim of this study is to determine the serum levels of these biomarkers in this population.

**Methods:**

This was a cross-sectional study where we randomly sampled 73 stored serum samples of PLWH who were enrolled in the Infectious Diseases Institute (IDI) ART long term (ALT cohort). We measured serum levels of E-selectin and ESM-1 by ELISA. Data was summarized using median and interquartile range. Inferential statistics were performed to determine predictors of elevated levels of E-selectin.

**Results:**

Of the 73 samples analyzed, 38 (52.1%) were from female participants. The mean age was 54 ± 9.0 years. Twenty participants (27.4%) had a history of smoking while 52 (71.2%) had a history of alcohol intake. Twenty-five (34.3%) of the participants were overweight whereas 4 (5.6%) were obese. Fifty-four (74%) had an undetectable viral load (≤ 0 copies/ml) and the mean duration of ART at the time of sampling (2014/2015) was 10.4 ± 0.4 years. While serum levels of ESM-1 were not detectable in any of our samples, the median E-selectin levels was 147.6 μm/L ranging from 8.44 μm/L and 1,979.36 μm/L. Sixty-seven participants (91.8%) had elevated levels of E-selectin (> 39 μm/L). CD4 count > 500 cells/µl compared to lower counts was a predictor of elevated levels of E-Selectin (adjusted Odd Ratio 12.5, 95% CI (1.03 — 149.95, p < 0.05).

**Conclusions:**

The majority (91.8%) of PLWH on long term ART had elevated levels of E-selectin. Having high CD4 count (> 500 cells/µl) was predictive of elevated levels of E-Selectin. Future work should longitudinally assess the trend of levels of E-selectin and ESM-1 while assessing for cardiovascular diseases endpoint.

**Supplementary Information:**

The online version contains supplementary material available at 10.1186/s12981-023-00519-x.

## Introduction

The prolonged life course of people living with HIV (PLWH) has contributed to the increase in the incidence and prevalence of cardiovascular diseases in this population [1–3]. In 2015, the global population-attributable fraction for cardiovascular disease associated with HIV infection was estimated at 0.92% and the disability adjusted life years (DALYS) of HIV associated cardiovascular diseases had increased by more than 3-fold from 0.74 million in 1990 to 2.57 million in 2015 with a significant proportion found in Sub Saharan Africa (SSA) [3]. Furthermore, a modelling study projected that 78% of PLWH will have been diagnosed with a cardiovascular disease by 2030 [4] which undermines the success made in curtailing the burden of HIV/AIDS.

Endothelial dysfunction is one of the proposed mechanism contributing to the increasing incidence of cardiovascular diseases in the HIV population [5]. Endothelial cell activation that precedes endothelial dysfunction is reportedly caused by multiple factors including persistent HIV induced immune activation, certain classes of ART such as nucleoside reverse transcriptase inhibitors and traditional cardiovascular risk factors such as smoking [6–8]. It is characterised by the increased expression of cell adhesion molecules such as intercellular adhesion molecule 1 (ICAM-1), vascular cell adhesion molecule 1 (VCAM-1) and selectins (P- or E-selectin) [9]. These molecules have been studied and their concentration has been shown to be raised among PLWH [10–14] with a high risk of cardiovascular diseases [15].

E- Selectin and endothelial cell-specific molecule-1 (ESM-1), also known as endocan-1 are highly specific to the endothelium since they are only produced by endothelial cells [16, 17] and are reliable markers of endothelial activation [18, 19]. Prior literature on the concentration of E-Selectin of PLWH on ART is inconsistent with some studies showing that initiation of ART normalized or significantly reduced the concentration of E-Selectin [20] while others found no difference in the concentration of E-Selectin before or after initiation of ART [21]. However, in PLWH on long term suppressive ART, E-Selectin was elevated [10, 14] which may imply that prolonged exposure to ART may cause damage to the endothelium. There is limited literature on the serum concentration of ESM-1 in PLWH in Sub-Saharan African, but a meta- analysis showed that elevated serum ESM-1 levels were significantly associated with cardiovascular diseases and could be one of the risk factors for cardiovascular diseases [19]. The absence of data on the serum concentration of ESM-1 in PLWH and the inconsistence in data on serum concentration of E-selectin among PLWH on long term ART (≥ 10 years) in SSA particularly Uganda provides the basis of our pilot study. In addition, we sought to determine the predictors of raised levels of E-Selectin and ESM-1.

## Methods

### Study design

This was a pilot cross-sectional study utilizing stored serum samples of 73 PLWH from the Infectious Diseases Institute (IDI) Antiretroviral therapy long-term (ALT) cohort.

### Study population

We retrieved frozen stored serum samples collected in 2014/2015 of 73 PLWH who were enrolled in the Long Term Antiretroviral (ALT) cohort. The ALT cohort is a prospective cohort of 1,000 PLWH who have been on ART for over 10 years and were followed up for an additional 10 years [22]. There was no formal sample size estimation done. However, the total number of participants was estimated based on the central limit theorem [23] with the assumption that recruitment of a minimum of 30 male and 30 female participants would give an approximate normal distribution of study variables. We excluded participants who were taking anticoagulants, steroids, statins, or any anti-inflammatory medication. In addition, participants with any known chronic illness such as hypertension, diabetes mellitus or who did not consent to the utilizations of their samples for other sub studies and with untraceable samples were excluded.

### Sampling technique

An electronic register of eligible participants in the ALT cohort was obtained and used to perform random sampling using computer generated random numbers.

### Study procedure

#### Participant data extraction

Deidentified clinical data was extracted from the participants’ charts. The data included: age, sex, duration of ART at sample collection, body mass index (BMI), history of smoking or alcohol intake, viral load, and CD4 count at sample collection.

#### Experimental procedures

Aliquots of the selected participants were retrieved from the IDI biorepository where they were stored at -80^0^ C since 2014/2015. The aliquots were then transported to the Makerere University Immunology laboratory working area within one hour. The serum was thawed and prepared for the Enzyme Linked Immunosorbent Assay tests (ELISA). The ELISA experiments were conducted by a trained laboratory immunologist.

E-Selectin: We assessed for serum levels of E-Selectin using commercially available ELISA kit, (catalogue number, abx050054) manufactured and distributed by Abbexa Limited, Cambridge Science Park, Cambridge, United Kingdom. We followed the manufacturer’s issued standard operating procedures (SOPs) to carry out sandwich ELISA experiment and concentration of E-Selectin above 39 μm /ml which was the lower Level of detection were considered elevated. Since E-Selectin is undetectable in unstimulated endothelial cell [24], we considered any detectable concentration of E-Selectin was elevated.

ESM-1: We used a commercially available kit (catalogue number abx151452) which was manufactured and distributed by Abbexa Limited, Cambridge Science Park, Cambridge, United Kingdom. The experiments were done following the manufacturer’s issued standard operating procedures (SOPs) and concentrations of ESM1 greater than 0.156 ng/ml, lower Level of detection were considered raised. We considered that cut-off because high and detectable ESM-1 concentration was associated with poor clinical outcomes [25, 26].

### Data analysis

The data was cleaned and exported into STATA 16.0, StataCorp LLC, College Station, Texas, USA for analysis. Normally distributed continuous variables were summarized using means and standard deviation and categorical variables were summarized using proportions and percentage. BMI was categorised as underweight (≤ 18.4 kg/m^2^), normal (18.5 — 24.9) kg/m^2^, overweight (25.0 — 29.9) kg/m^2^ and obese (≥ 30) kg/m^2^ [27] while history of smoking and alcohol intake was categorized as yes or no. Age was further categorized as < 44 years, 45.0 — 65, and > 65 years. Comparisons between the continuous variables were done using the student’s t-test and Mann Whitney U test. while the chi-square and Fishers’ Exact tests were used for the categorical variables. Box plots were used for data visualization of the concentrations of E-Selectin and Viral load. The proportion of PWLH with elevated E-selectin levels was summarized as a proportion with its 95% confidence interval while no PLWH had detectable levels of ESM-1.

For the regression analysis, the outcome variable of elevated E-Selectin levels was dichotomized as 1 “yes elevated E-selectin levels (> 39)” 0” No elevated E-selectin levels (≤ 39)”. We conducted a univariate analysis for the independent variables and the outcome. In the multivariable logistic regression analysis, we used the backward and forward selection methods where variables with a p value < 0.25 or previously known in the literature to induce endothelial activation were considered (i.e., sex, body mass index, age, CD4 count, smoking and alcohol intake status). We assessed interaction by forming two-way interaction terms and performed and likelihood ratio tests between sex and CD4, alcohol use, BMI. We assessed confounding by considering a 10% or more change in the odds ratio with a model with the variable and one without. The goodness of fit of the model was assessed using the Hosmer-Lemeshow goodness of fit test. The odds ratios (OR) with their 95% Confidence Interval (CI) are presented with a p-value of < 0.05 considered statistically significant.

## Results

### Socio-demographics and clinical characteristics

Of the 73 participants included into the study, 38 (52.1%) were female. The mean age of the participants was 54 ± 9 years. Thirty-six (49.3%) participants were married, and 58 (79.5%) were employed. Regarding their clinical history (Table 1), 19 (26%) had detectable viral load (> 0 copies/ml) while only five (6.9%%) had CD4 count of less than 200 cells/µL. Four (5.6%) were obese. The mean duration on ART at the time of sampling (2014/2015) was 10.4 ± 0.4 and 22 (30.1%) of the participants were on a Tenofovir (TDF) based regimen. Twenty (27.4%) had a history of smoking while 52 (71.2%) had history of alcohol use.

**Table 1:** The socio-demographic and clinical Characteristics of PLWH in the ALT cohort at IDI, Kampala who took part in our study

Characteristic	Freq.	Percentage
**Socio-demographics**		
**Sex**		
Female	38	52.1
Male	35	47.9
**Age**		
Mean ± standard deviation (SD)	53.8 ± 9	
**Age categories**		
< 45 years	10	13.7
45 — 65 years	55	75.3
> 65 years	8	11.0
**Employment status**		
No	15	20.6
Yes	58	79.5
Monthly income*		
Less than 14 USD	13	18.8
14–27 USD	11	15.9
28–139 USD	33	47.81
140–279 USD	11	15.9
Above 279	1	1.4
**Clinical Characteristics**		
**Body mass index (BMI)**		
Normal, 18.5–24.9 kg/m^2^	42	58.3
Underweight, < 18.5 kg/m^2^	11	15.3
Overweight, 25–30 kg/m^2^	15	20.8
Obese, > 30 kg/m^2^	4	5.6
**History of smoking**		
Yes	20	27.4
No	52	71.2
Undefined	1	1.4
**History of Alcohol intake**		
Yes	52	71.2
No	20	27.4
Undefined	1	1.4
**Viral load**		
Detected (> 0 copies/ml)	19	26.0
Undetectable (< 0 copies/ml)	54	74.0
**CD4 count at sample collection**		
Less than 500 cells/µl	38	52.1
Above 500 cells/µl	35	47.9
Duration of ART at sample collection**		
Mean ± SD	10.4 ± 0.4	
**ARV regimen at sample collection (2014/2015)**		
ZDV-3TC-NVP	35	48.0
ZDV-3TC-EFV	16	22.9
TDF-3TC-NVP	12	16.4
TDF-3TC-EFV	3	4.1
TDF-3TC-ATV/r	3	4.1
TDF-3TC-LPV/r	3	4.1
TDF-FTC-LPV/r	1	1.4
ARV regimen 1 (2004/2005) **		
d4T (30)-3TC-NVP	51	76.1
d4T (40)-3TC-NVP	14	20.9
ZDV-3TC-NVP	1	1.5
ZDV-3TC-EFV	1	1.5
Reason ever switched from first line **		
Due to new TB	7	10.5
MoH recommendation	40	59.7
Other (Specify)	1	1.5
Pregnancy or risk of pregnancy	1	1.5
Toxicity/complications	16	23.9
Virologic failure	2	3

*Four [4] participants had no income recorded. **Six [6] participants had missing data on ART start date

### Biomarkers of endothelial cell activation

We found that 67 (91.8% CI (82.6–96.3)) had elevated serum levels of E-Selectin (Table 2). The median concentration of E-selectin in serum was 147.56 μm/L with the 8.44 μm/L as the minimum concentration while 1,979.4 μm/L as the maximum concentration. There were no detectable levels of ESM-1 hence this biomarker was dropped during analysis.

**Table 2:** The serum concentration of E-Selectin and variation in the concentration of E-Selectin by sex, history of smoking and BMI category of PLWH in the ALT Cohort at IDI, Kampala, Uganda

Characteristic			
Median concentration of E-Selectin, (Ranges)	147.6 (8.4 — 1,979.4)		
	**n (%)**	**95% Confidence Interval**	
E-Selectin concentration ≤ 39 μm/L	6 (8.2)	3.67 — 17.4	
E-Selectin concentration > 39 μm/L	67 (91.8)	82.6 — 96.3	
**Variation in the concentration of E-Selectin by different variables**			
	**≤ 39.0 μm/L, n (%)**	**> 39.0 μm/L and** **above, n (%)**	**P value based on Fisher’s exact tests**
**Sex**			
Male	3 (7.9)	35 (92.1)	
Female	3 (8.6)	32 (91.4)	1.0
**History of smoking**			
Undefined	0 (0.0)	1 (100.0)	
Yes	1 (5.0)	19(95.0)	
No	5 (9.6)	47 (90.4)	0.6
**BMI**			
Normal, 18.5–24.9 kg/m^2^	2(5.1)	37 (94.9)	
Underweight, < 18.5 kg/m^2^	1 (9.1)	10 (90.9)	
Overweight, 25–30 kg/m^2^	3 (16.7)	15 (83.3)	
Obese, > 30 kg/m^2^	0 (0.0)	4 (100.0)	0.5

*BMI, Body Mass Index*

### Predictors of elevated serum levels of E-selectin

**Fig. 1:**
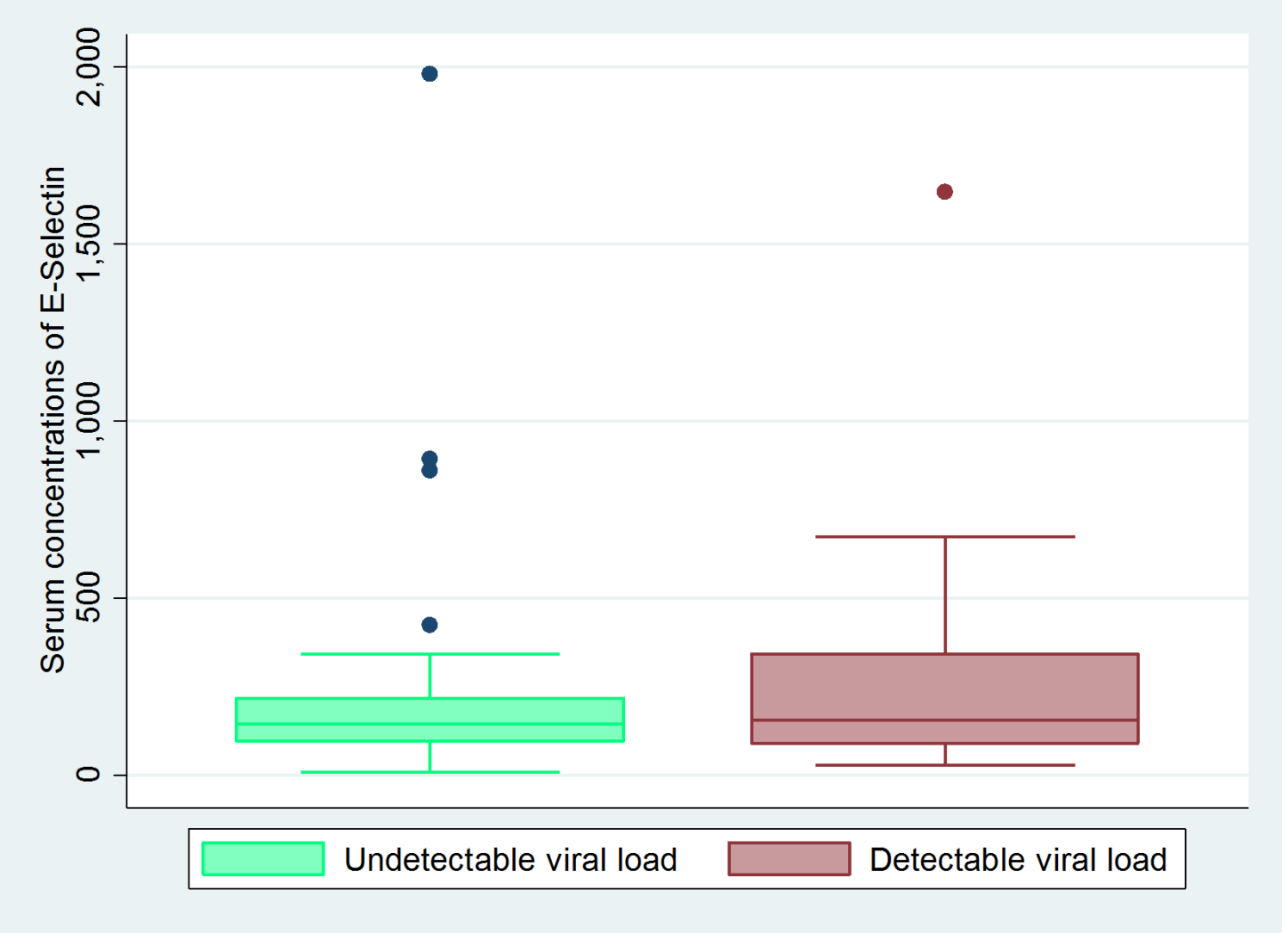
The relationship between E-Selectin concentration and viral load categories of PLWH in the ALT Cohort at IDI. The X - axis is the concentration of E-Selectin while the Y - axis is the viral load concentration. Mann Whitney U test, p value = 0.646

In the bivariate analysis (Table 2), sex (p value < 0.25 ), viral load (p value = 0.65) (Fig. 1), history of smoking (p value = 0.6) and being obese (p value = 0.5) were not significant.

In the multivariate analysis (Table 3), participants who had a CD4 count of more than 500 cells/µl were 12.5 times likely to have elevated serum concentrations of E-Selectin (adjusted OR: 12.5, 95% CI (1.03 — 149.95, p value = 0.047). Being above 45 years old (adjusted OR: 1, 95% CI (0.07 — 13.99), p value > 0.05 ), underweight (adjusted OR: 0.8, 95% CI (0.06 — 10.6), p value > 0.05), overweight or obese (adjusted OR: 0.1, 95% CI (0 — 1.2), p value = 0.08) and having a history of alcohol intake (adjusted OR: 6.6 95% CI (0.5 — 84.5), p value > 0.05) were not associated with raised serum concentrations of E-Selectin.

**Table 3:** The predictors of elevated serum levels of E-Selectin of PLWH in ALT cohort at IDI, Kampala

Characteristics	≤ 39 μm/L, n (%)	> 39 μm/L, n (%)	Crude OR, (95%CI)	P value	Adjusted OR (95% CI)	P value
**Sex**						
Female	3 (50.0)	35 (52.2)	1			
Male	3 (50.0)	32 (47.8)	0.914 (0.172–4.86)	0.47		
**Age**						
less than 45 years	1 (176.7)	9 (13.4)	1		1	
45 years and above	5 (83.3)	58 (86.6)	1.29 (0.134–12.338)	0.83	1 (0.075–13.99)	0.99
**Viral load**						
Not detected (< 0 copies per ml)	4 (66.7)	50 (74.6)	1			
Detectable (> 0 copies/ml)	2 (33.3)	17 (25.4)	0.68 (0.1–4.1)	0.67		
**CD4 count at sample collection**						
Above 500 cells/µl	1 (16.7)	34 (50.8)	5.12 (0.6–46.4)	0.14	12.5 (1.03- 149.95)	0.047
**BMI**						
Normal, 18.5–24.9 kg/m^2^	2 (33.3)	37 (56.1)	1		1	
Underweight, < 18.5 kg/m^2^	1 (16.7)	10 (15.2)	0.54 (0.044–6.585)	0.63	0.8 (0.1–10.5)	0.76
Overweight/obese, > 25 kg/m^2^	3 (50.0)	19 (28.8)	0.34 (0.052–2.27)	0.26	0.1 (0. -1.2)	0.08
**History of smoking**						
No/ undefined	1 (16.7)	19 (28.4)	1			
Yes	5 (83.3)	48 (71.6)	1.98 (0.217–18.073)	0.54		
**History of alcohol intake**						
No/ Unknown	5 (83.3)	47 (70.2)	1		1	
Yes	1(16.7)	20 (29.8)	0.47 (0.052–4.284)	0.50	6.6 (0.5–84.5)	0.15

## Discussion

In our pilot laboratory based cross-sectional study, we found that close to 92% of PLWH on long term ART had raised serum levels of E-selectin while none of them had detectable levels of ESM-1. CD4 count > 500 cells/µl was a predictor of increased concentration of E-Selectin.

Prior studies investigating the concentration of E-Selectin in PLWH with a median duration of ART of 8.5 ± 2.7 years [10] and 12 years [14] reported that all participants had elevated E-Selectin levels above the level of detection which is consistent with our findings. HIV induced proteins such as TAT have been reported to upregulate E-selectin causing endothelial activation which plays a key role in leukocyte adhesion and migration [28]. In addition, HIV induces the production of proinflammatory cytokines such as Tumor Necrosis Factor (TNF-α) and reactive oxygen species which may directly or indirectly cause an increase in the expression of E-Selectin subsequently leading to endothelial activation [5, 29]. Another possible explanation for the detectable E-selectin levels is the use of Nucleoside Reverse Transcriptase Inhibitors (NRTIs) to which our participants had prior exposure to. Experimental studies suggest that sidovudine and stavudine precipitated the production of reactive oxygen species (ROS) such as superoxide which cause direct damage to the endothelium [7, 30, 31]. NRTI are not prescribed as a monotherapy but in combination with other ART classes such as Protease inhibitors (PI) whose endothelial effects are well documented [32]. The synergistic effects of chronic HIV infection and long-term ART could be responsible for the increased serum levels of E-Selectin in our cohort.

We found no detectable serum levels of ESM-1. To the best of our knowledge, there are no studies investigating the relationship between HIV, ART, and ESM-1. However, the concentration of ESM-1 is reportedly increased during chronic diseases such as Coronary Artery disease [33], chronic inflammatory conditions such as Systemic Lupus Erythematous [34] and acute severe infections such as sepsis [35, 36]. In addition, Kazunori and colleagues found that ESM-1 levels were elevated at the onset of the bacterial infection but subsequently reduced [35]. A possible explanation for the finding in our study could be that ESM-1 may be elevated in acute HIV phase or in patients with HIV who are not on ART, but not PLWH who have been on long term suppressive ART like the participants in our study. An alternative underlying mechanism stems from the supposedly pro-inflammatory effects of ESM-1 which involves the upregulation of endothelial adhesion molecules such as E-Selectin, VCAM-1, and ICAM-1 [17] that are elevated in PWH on long term ART [10, 11, 14]. The presence of these molecules might exert negative feedback on production of ESM-1 by downregulating its expression. One additional potential explanation for the absence of ESM-1 in our study may be attributed to the breakdown of ESM-1 protein because of long-term storage. More mechanistic studies investigating the relationship between HIV, ART and ESM-1 ought to be carried as this could be provide a possible target that can be used to predict or modulate endothelial cell activation and subsequently atherosclerosis.

Unlike other studies that have shown that low CD4 count is associated with endothelial dysfunction [37, 38], our study reported the contrary. This could be attributed to the different parameters to assess for endothelial dysfunction and their inclusion of participants with comorbidities. For example, Ho and colleagues found that nadir CD4 count of < 350 cells/µl was associated with endothelial dysfunction measured using flow mediated dilation (Beta coefficient of -1.22, 95% CI (-2.20 to -0.19). We postulate that PLWH were aggressively treated with ART to achieve the high CD4 counts. This exposed their endothelium to the unwanted effects of ART hence the increased risk to raised levels of E-Selectin. In this study, history of smoking, history of alcohol intake, viral load, and age which are risk factors of atherosclerotic cardiovascular disease among PLWH were not predictors of raised levels of E-Selectin which is consistent with findings from a study conducted in Botswana [10].

Our study had a few limitations. Our sample size was small which may have affected our statistical power and the extensive analysis of potential confounders. We also did not compare with a healthy control population. However, our study has some strengths which include generating significant findings which can be used to design future studies. Furthermore, we considered participants without pre-existing chronic illness such as diabetes mellitus or inflammatory conditions enabling to eliminate some of the potential confounders.

## Conclusion

In our cross-sectional study, we found that most (91.8%) of the sampled participants had raised serum levels of E-Selectin while none had detectable serum levels of ESM-1. High CD4 count was significantly predictive of elevated levels of E-Selectin. More studies should investigation the relationship between HIV, ART, E-Selectin and ESM-1 using a larger sample size and with clinical endpoint.

## Electronic supplementary material

Below is the link to the electronic supplementary material.

**Additional file 1:** Table S1: The raw results of ESM-1 ELISA experiment.

## Data Availability

The datasets generated and analyzed during the current study are not publicly available due to ethical restrictions regarding patient data but are available on submission of a formal request to the investigators committee of the ALT cohort, bcastelnuovo@idi.co.ug. The raw results from the laboratory experiments have been included as supplementary file 1.

## Abbreviations

ART: Antiretroviral Therapy
ALT: ART long term
ESM-1: Endothelial cell-specific molecule-1
ELISA: Enzyme linked Immunosorbent Assay
IDI: Infectious Diseases Institute
ICAM: Intercellular Cell Adhesion Molecule
JCRC: Joint Clinical Research Center
LMIC: Low- and Middle-Income Countries
NRTI: Nucleoside Reverse Transcriptase Inhibitor
PI: Protease Inhibitor
PWH: People With HIV
VCAM: Vascular cell adhesion molecule 1
SSA: Sub-Saharan Africa
UNCST: Uganda National Council for Science and Technology
