# Supplementary material for: Serum E-selectin and endothelial cell-specific Molecule-1 levels among people living with HIV on long term ART in Uganda: a pilot cross-sectional study
Source: AIDS Res Ther. 2023 May 9;20:26. doi: 10.1186/s12981-023-00519-x (PMC10169468; doi:10.1186/s12981-023-00519-x)
Supplement: Supplementary file 1 — Additional file 1: Table S1: The raw results of ESM-1 ELISA experiment. [file 12981_2023_519_MOESM1_ESM.docx]

Supplementary File_1

Table 1: The raw results of ESM-1 ELISA experiment.

| **Sample Number** | **Concentration** |
| --- | --- |
| Blank | <0.000 |
| Blank-R | <0.000 |
| S2 | 7.9 |
| S1R | 9.874 |
| S3 | 5.763 |
| S2R | 7.651 |
| S4 | 3.041 |
| S3R | 4.162 |
| S5 | 1.13 |
| S4R | 1.995 |
| S6 | 0.209 |
| S5R | 0.522 |
| S7 | <0.000 |
| S6R | <0.000 |
| S8 | <0.000 |
| S7R | <0.000 |
| 1 | <0.000 |
| 2 | <0.000 |
| 3 | <0.000 |
| 4 | <0.000 |
| 5 | <0.000 |
| 6 | <0.000 |
| 7 | <0.000 |
| 8 | <0.000 |
| 9 | <0.000 |
| 10 | <0.000 |
| 11 | <0.000 |
| 12 | <0.000 |
| 13 | <0.000 |
| 14 | <0.000 |
| 15 | <0.000 |
| 16 | <0.000 |
| 17 | <0.000 |
| 18 | <0.000 |
| 19 | <0.000 |
| 20 | <0.000 |
| 21 | <0.000 |
| 22 | <0.000 |
| 23 | <0.000 |
| 24 | <0.000 |
| 25 | <0.000 |
| 26 | <0.000 |
| 27 | <0.000 |
| 28 | <0.000 |
| 29 | <0.000 |
| 30 | <0.000 |
| 31 | <0.000 |
| 32 | <0.000 |
| 33 | <0.000 |
| 34 | <0.000 |
| 35 | <0.000 |
| 36 | <0.000 |
| 37 | <0.000 |
| 38 | <0.000 |
| 39 | <0.000 |
| 40 | <0.000 |
| 41 | <0.000 |
| 42 | <0.000 |
| 43 | <0.000 |
| 44 | <0.000 |
| 45 | <0.000 |
| 46 | <0.000 |
| 47 | <0.000 |
| 48 | <0.000 |
| 49 | <0.000 |
| 50 | <0.000 |
| 51 | <0.000 |
| 52 | <0.000 |
| 53 | <0.000 |
| 54 | <0.000 |
| 55 | <0.000 |
| 56 | <0.000 |
| 57 | <0.000 |
| 58 | <0.000 |
| 59 | <0.000 |
| 60 | <0.000 |
| 61 | <0.000 |
| 62 | <0.000 |
| 63 | <0.000 |
| 64 | <0.000 |
| 65 | <0.000 |
| 66 | <0.000 |
| 67 | <0.000 |
| 68 | <0.000 |
| 69 | <0.000 |
| 70 | <0.000 |
| 71 | <0.000 |
| 72 | <0.000 |
| 73 | <0.000 |
| 74 | <0.000 |
| 75 | <0.000 |
| 76 | <0.000 |
| 77 | <0.000 |
| 78 | <0.000 |
| 79 | <0.000 |
| 80 | <0.000 |

Table 2: The raw results from the E-Selectin ELISA experiment.

| **Sample Number** | **Concentration** |
| --- | --- |
| Blank | <0.000 |
| Blank | 20.883 |
| S2 | 2523.98 |
| S1R | 2419.92 |
| S3 | 1193.255 |
| S2R | 1353.304 |
| S4 | 798.506 |
| S3R | 613.007 |
| S5 | 306.483 |
| S4R | 284.992 |
| S6 | 167.924 |
| S5R | 108.542 |
| S7 | 149.827 |
| S6R | 17.49 |
| S8 | <0.000 |
| S7R | <0.000 |
| 1 | 75.741 |
| 2 | 128.902 |
| 3 | 62.733 |
| 4 | 214.864 |
| 5 | 246.535 |
| 6 | 168.49 |
| 7 | 187.153 |
| 8 | 31.628 |
| 9 | 140.213 |
| 10 | 84.224 |
| 11 | 91.01 |
| 12 | 165.097 |
| 13 | 86.486 |
| 14 | 248.231 |
| 15 | 217.692 |
| 16 | 85.355 |
| 17 | 300.262 |
| 18 | 337.022 |
| 19 | 505.554 |
| 20 | 108.542 |
| 21 | 137.385 |
| 22 | 87.051 |
| 23 | 342.677 |
| 24 | 94.404 |
| 25 | <0.000 |
| 26 | 28.8 |
| 27 | 115.329 |
| 28 | 341.546 |
| 29 | 114.763 |
| 30 | 255.018 |
| 31 | 59.905 |
| 32 | 186.587 |
| 33 | 161.138 |
| 34 | 147.565 |
| 35 | 187.153 |
| 36 | 179.801 |
| 37 | 87.051 |
| 38 | 127.771 |
| 39 | 214.864 |
| 40 | 16.924 |
| 41 | 121.55 |
| 42 | 238.052 |
| 43 | 424.116 |
| 44 | 1647.387 |
| 45 | 672.955 |
| 46 | 103.452 |
| 47 | 156.613 |
| 48 | 149.827 |
| 49 | 71.216 |
| 50 | 209.209 |
| 51 | 352.292 |
| 52 | 252.756 |
| 53 | 116.46 |
| 54 | 8.441 |
| 55 | 104.018 |
| 56 | 81.396 |
| 57 | 35.021 |
| 58 | 93.838 |
| 59 | 211.471 |
| 60 | 239.183 |
| 61 | 117.591 |
| 62 | 1979.361 |
| 63 | 191.112 |
| 64 | 113.632 |
| 65 | 60.471 |
| 66 | 282.73 |
| 67 | 169.055 |
| 68 | 140.778 |
| 69 | 196.676 |
| 70 | 81.962 |
| 71 | 217.127 |
| 72 | 105.149 |
| 73 | 127.205 |
| 74 | 142.475 |
| 75 | 36.153 |
| 76 | 115.329 |
| 77 | 287.254 |
| 78 | 859.584 |
| 79 | 892.386 |
